# Characterization of T-bet expressing B cells in lupus patients indicates a putative prognostic and therapeutic value of these cells for the disease

**DOI:** 10.1093/cei/uxaf008

**Published:** 2025-02-07

**Authors:** Athanasios Sachinidis, Maria Trachana, Anna Taparkou, George Gavriilidis, Vasileios Vasileiou, Sofoklis Keisaris, Panayotis Verginis, Christina Adamichou, Dimitrios Boumpas, Fotis Psomopoulos, Alexandros Garyfallos

**Affiliations:** 4th Department of Internal Medicine, Hippokration General Hospital, School of Medicine, Aristotle University of Thessaloniki, Thessaloniki, Greece; Paediatric Immunology and Rheumatology Referral Centre, 1st Paediatric Department, Hippokration General Hospital, School of Medicine, Aristotle University of Thessaloniki, Thessaloniki, Greece; Paediatric Immunology and Rheumatology Referral Centre, 1st Paediatric Department, Hippokration General Hospital, School of Medicine, Aristotle University of Thessaloniki, Thessaloniki, Greece; Institute of Applied Biosciences, Centre for Research and Technology Hellas, Thermi, Thessaloniki, Greece; Institute of Applied Biosciences, Centre for Research and Technology Hellas, Thermi, Thessaloniki, Greece; Institute of Applied Biosciences, Centre for Research and Technology Hellas, Thermi, Thessaloniki, Greece; Laboratory of Immune Regulation and Tolerance, Division of Basic Sciences, Medical School, University of Crete, Heraklion, Greece; 4th Department of Internal Medicine, Hippokration General Hospital, School of Medicine, Aristotle University of Thessaloniki, Thessaloniki, Greece; 4th Department of Internal Medicine, “Attikon” University Hospital, National and Kapodistrian University of Athens, Athens, Greece; Institute of Applied Biosciences, Centre for Research and Technology Hellas, Thermi, Thessaloniki, Greece; 4th Department of Internal Medicine, Hippokration General Hospital, School of Medicine, Aristotle University of Thessaloniki, Thessaloniki, Greece

**Keywords:** age-associated B cells, double-negative B cells, DN2, T-bet+ B cells, systemic lupus erythematosus

## Abstract

**Objective:**

To investigate whether T-bet+ B cells, as well as age-associated B cells/ABCs (CD19 + CD21-CD11c + T-bet+) and double-negative B cells/DN (CD19 + IgD-CD27- CXCR5-T-bet+), serve as prognostic and/or therapeutic tools for systemic lupus erythematosus (SLE) in humans.

**Methods:**

Flow cytometry was used for enumerating T-bet+ B cells and ABCs/DN subsets, found in the peripheral blood of 10 healthy donors and 22 active SLE patients. Whole blood assay cultures, combined with *in vitro* pharmacological treatments, were performed to evaluate the effects of hydroxychloroquine, anifrolumab, and fasudil (a ROCK kinase inhibitor) on T-bet+ B cells’ percentage. Moreover, previously published single-cell RNA sequencing (scRNA-seq) data were used in a meta-analysis to allow characterization of genes and pathways associated with the biology of T-bet in B cells.

**Results:**

T-bet+ B cells displayed an expansion in SLE patients [1.47 (1.9–0.7) vs 10.85 (37.4–3.6)]. Similarly, both ABCs and DN were found to be expanded. Interestingly, percentages of T-bet+ B cells positively correlated with patients’ SLEDAI scores (rs = 0.55, *P* = 0.007). Cell culture experiments conducted revealed that all three agents tested can deplete T-bet + B cells (without affecting the cell viability of lymphocytes, T cells, and B cells). According to bioinformatics analyses, T-bet is highly expressed in two B-cell clusters with pathogenic characteristics for SLE (designated as atypical memory B cells and activated naïve B cells). These clusters can be targeted for therapeutic interventions.

**Conclusions:**

T-bet+ B cells can serve as a putative prognostic biomarker of lupus severity. Circumstantial data suggest that these cells may promote disease pathogenesis and may represent a novel therapeutic target.

## Teaser

This paper investigates whether T-bet+ B cells, as well as age-associated B cells/ABCs (CD19+CD21-CD11c+T-bet+) and double-negative B cells/DN (CD19+IgD-CD27-CXCR5-T-bet+), serve as prognostic and/or therapeutic tools for systemic lupus erythematosus (SLE) in humans. Flow cytometry was used for enumerating T-bet+ B cells and ABCs/DN subsets, found in the peripheral blood of 10 healthy donors and 22 active SLE patients.

## Graphical Abstract

**Graphical Abstract:**
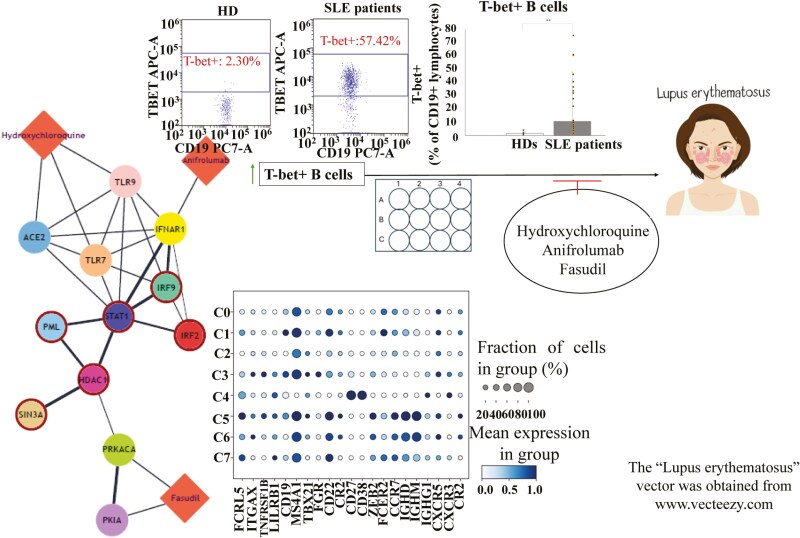
**Graphical abstract caption.** Flow cytometry experiments, in conjunction with *in vitro* and *in silico* analyses, have shed light on putative prognostic and therapeutic values of T-bet + B cells in SLE.

## Introduction

Despite being a Th1 lineage commitment regulator, transcription factor T-bet (encoded by *TBX21* gene) is also expressed in other immune cell types, such as dendritic cells, natural killer cells, and B cells [1]. In the case of B cells, more specifically, the transcription factor seems to regulate their isotype switching to IgG2a/c in mice and IgG1/3 in humans [2].

Sub-populations of T-bet+ B cells, known in the literature as age-associated B cells/ABCs (CD19^+^CD21^−^CD11c^+^T-bet^+^) and/or double-negative B cells/DN (CD19^+^IgD^−^CD27^−^CXCR5^−^T-bet^+^), expand in various autoimmune diseases, including systemic lupus erythematosus (SLE), and drive their pathogenesis [3, 4]. Interestingly, the elevated expression of T-bet in these cell populations has been associated with production of autoantibodies, enhanced antigen presentation to T cells, and also formation of spontaneous germinal centers. Due to these reasons, T-bet+ B cells have been considered as potential therapeutic targets for autoimmune diseases [5].

It is important to mention that the induction of T-bet in ABCs/DN derives from the synergistic triggering of B-cell receptor (BCR), Toll-like receptor 7 (TLR7) or TLR9 and IFNγR or IL-21R [5]. The signals of IL-21 and IFNγ probably stem from T follicular helper cells (Tfh) [5, 6]. The cooperation of these two cytokines leads to ABC/DN expansion and promotes their differentiation into antibody-secreting cells (ASCs) [3, 4].

Currently, the exact role and functions of ABCs/DN in the pathogenesis of autoimmunity are not completely understood. Furthermore, the importance of T-bet for ABC/DN biology has been questioned, as functional murine ABCs had been generated, both *in vitro* and *in vivo*, in the absence of its expression in B cells [7]. In addition to this, a recently published study has indicated that transcription factor ZEB2 is more important than T-bet for ABC specification and differentiation, both in mice and humans [8]. Although we do not ignore these data, we cannot also ignore data that indicate an important role for T-bet+ B cells in autoimmunity pathogenesis and therapy [5, 9]. To this end, in our study, we have put an emphasis on T-bet and aimed to clarify whether T-bet+ B cells, as well as the subsets of ABCs and DN, serve as prognostic and/or therapeutic tools for SLE in humans.

## Materials and methods

### 2.1 Healthy donors (HDs) and SLE patients

We have enrolled 10 HDs (staff of the 4th Department of Internal Medicine in Hippokration General Hospital of Thessaloniki) and 22 active SLE patients (17 from the aforementioned clinic and 5 more from the 1st Department of Nephrology in the Hippokration Hospital). All patients fulfilled the EULAR/ACR 2019 criteria [10]. From each individual, we have obtained a blood sample and a serum sample, for flow cytometry and ELISA experiments, respectively. Along with the samples, we collected data regarding the clinical and/or laboratory characteristics of the subjects, so as to identify correlations between their profiles and T-bet+ B cell populations. The inclusion and exclusion criteria for the participants are reported in the Supplementary (**S1**).

### Flow cytometry

Flow cytometry was used for the enumeration of T-bet+ B cells and ABC/DN subsets, found in the peripheral blood of the subjects. Two panels of conjugated antibodies were used, first one referring to ABCs (CD45^+^CD19^+^CD11c^+^CD21^−^T-bet^+^) and second one to DN/DN2 B cells (CD45^+^CD19^+^IgD^−^CD27^−^CXCR5^−^T-bet^+^). BriCyte E6 flow cytometer (Mindray) was used for all the experiments. Prior to the enumeration of T-bet expressing B-cell populations, the main populations of lymphocytes (CD45^+^CD3^+^ and CD45 + CD19^+^ cells) had also been enumerated, so as to evaluate the general condition of each individual. Of note, the gating strategy and the antibodies and reagents used are all presented in the Supplementary (**S2**, **S3**).

### Whole blood assay—cell culture

In order to evaluate *in vitro* the effects of some pharmacological agents on T-bet + B cells, we conducted cell cultures of whole blood, known as whole blood assay (WBA). WBA is a reliable *in vitro* method to access human immune responses that also provides a more physiological environment, which mimics the human blood condition better than peripheral blood mononuclear cell (PBMC) and/or B cell cultures [11, 12]. For the procedure, we used 12-well cell culture plates and seeded 10^6 cells/ml, in each well. The medium used was RPMI 1640 (Thermo Fischer Scientific, cat. #22400097), enriched with 1% penicillin-streptomycin (Thermo Fisher Scientific, cat. #15070063). The blood donor, for all WBAs conducted, was the same person (a 55-year-old female HD). The blood was collected via heparinized syringe.

### Induction of T-bet in B cells cultured

Since we wanted to evaluate the effects of pharmaceutical agents on T-bet+ B cells, we had to induce the transcription factor first, in the B cells cultured, so as to create a lupus-like environment. The blood donor for the cultures, as mentioned above, was a 55-year-old female HD, thus it was not expected for T-bet to be highly expressed in B cells, without any stimulation. For the induction, the cells were simultaneously treated with 1 μg/ml of a TLR7 agonist, known as R848 (InvivoGen, cat.# tltl-r848), 10 ng/ml of IFNγ (BioLegend, cat.#570204) and 10 ng/ml of IL-21 (BioLegend, cat.#571202). Obviously, unstimulated cells served as control. The incubation of the 12-well plate, following the addition of stimuli to the cells, took place in a proper incubator, under desired conditions (5% CO_2_ and 37 ^o^C), for 24 hours.

### Pharmacological treatments (*in vitro*)

The pharmaceutical agents tested were hydroxychloroquine (HCQ), anifrolumab, and a Rho-associated protein kinase (ROCK) inhibitor, known as fasudil. The first two drugs are used in the clinical practice for SLE cases [13, 14], while fasudil has been reported to attenuate the disease, following its administration in lupus-prone mice [15]. One hour before terminating the cultures, we (separately) treated the cells with pharmaceutical agents. We tested 1 μM, 10 μM and 50 μM of HCQ and fasudil, and 2 μg/ml, 10 μg/ml and 30 μg/ml of anifrolumab. The truth is that various concentrations of these three drugs have been tested *in vitro* by others and, after a literature review, we randomly selected the abovementioned concentrations for our experiments. We note that fasudil and anifrolumab had been purchased for research use (Sigma-Aldrich, cat.#CDS021620-10MG and Thermo Fischer Scientific, cat.#MA5-42010, respectively), while HCQ stock was made properly diluting a 200 mg pill of plaquenil (Sanofi) in sterile water. Fasudil was also diluted in sterile water, whereas anifrolumab was diluted in PBS 1X, according to the manufacturer’s guidelines.

### Cell culture toxicity tests—evaluation of cell viability

In order to evaluate the cell viability of the cultures, both before and after the stimulation of the cells and also the pharmacological treatments, we performed a flow cytometry 7-amino actinomycin D (7-AAD) staining protocol. This fluorescent chemical compound displays a strong affinity for DNA and is generally excluded from viable cells, as it is membrane impermeant [16]. The staining procedure was conducted according to the guidelines of the manufacturer of the product purchased (Beckman Coulter, cat. #A07704).

In addition to 7-AAD staining protocol, we performed cell culture toxicity tests by enumerating the main populations of lymphocytes (CD45^+^CD3^+^ and CD45 + CD19^+^ cells). Flow cytometry was used for the process.

### ELISA

ELISA was used for the estimation of IFNα and TNFα levels in the serum samples, derived from the participants of the study. The levels of IFNα had also been estimated in the supernatants of the cell cultures conducted. Of note, IFNα is one of the major cytokines associated with SLE pathogenesis [17], while TNFα is a pro-inflammatory cytokine that is secreted by ABCs [18]. Human IFNA1 Sandwich ELISA Kit (Proteintech, cat. #KE00044) was used for IFNα estimation and AuthentiKine^TM^ Human TNF-alpha Sandwich ELISA Kit (Proteintech, cat. #KE00154) was used for TNFα estimation. According to the manufacturer, the sensitivity of the kit regarding IFNα is 1.5pg/ml and the sensitivity of the TNFα kit is 8.6pg/ml. The process was based on the protocol suggested by the manufacturer of the kit products.

### Single-cell RNA sequencing (scRNA-seq) analysis

To glean some insights on the molecular circuitries governing T-bet+ B cells, we resorted to three publicly available scRNA-seq datasets (GSE162577, GSE142016, and GSE135779), all retrieved from Gene Expression Omnibus database [19]. The data analyzed refers to the PBMCs of 6 HDs and 11 SLE patients (all adult individuals). During quality control, cells with fewer than 500 or more than 6000 expressed genes, as well as more than 10% of mitochondrial genes, were removed from the analysis. Data was integrated via Seurat v.5 pipeline by deploying the “Harmony” algorithm [20, 21]. After quality control assessment and data pre-processing (including normalization/scaling, dimensionality reduction, clustering, integration, and batch effect correction), we compiled a unified single-cell object, encompassing various PBMC types (**S4**), and proceeded with a nested analysis focusing only on the B cells. We next probed for key marker genes (S5), which are indicative of discrete B-cell clusters, like naïve B cells, memory B cells, and/or ABCs/DN. In order to unravel molecular differences in T-bet^+^ B cells between HDs and SLE patients, we conducted a differential expression analysis via decoupleR package [22], to infer transcription factor (TF) and signaling pathway activities, as well as functional enrichment of biological terms. As far as TFs are concerned, we note that the terms “hyper-active” and/or “hypo-active,” used in the following sections of the manuscript, refer to the enrichment score of a TF, which actually serves as a signal for the role of a transcription factor in regulating genes of interest and thus being important—maybe a key regulator or a driver—for the biological process or condition being studied (which is SLE, in this case). In a similar manner, “hyper-activity” and/or “hypo-activity” of a signaling pathway are terms, indicative of the association of a pathway with the genes of interest and also of biological relevance to a condition. Lastly, STRING-db and DrugBank databases were used as bioinformatic resources for the construction of drug-gene bipartite networks [23, 24], with an aim to predict whether HCQ, anifrolumab and/or fasudil have an impact on the hyper-active TFs in the T-bet^hi^ B cell clusters, detected during our analysis.

### Statistical analysis

All statistical tests were performed in IBM SPSS Statistics for Windows, version 27. Shapiro-Wilk test was used for normality testing. Normally distributed variables were expressed as MEAN ± SD (standard deviation), whereas non-parametric variables were expressed as median and interquartile range (IQR: Q3—Q1). In case of significance, Mann–Whitney *U* test and Student’s *t* test (in some cases) were used. Correlations were assessed using Spearman’s rank correlation coefficient. As far as *P* values are concerned, *P* ≤ 0.05 (*) and *P* ≤ 0.01 (**) were considered as statistically significant.

## Results

### Demographics of the participants

We provide a table, regarding the demographics of all participants (Table 1). We note that according to Mann–Whitney *U* test, the difference between the age of HDs and the age of the patients is considered as statistically non-significant (*P* = 0.16).

**Table 1:** Demographics of HDs and SLE patients, enrolled for the study.

Demographics of healthy donors (HDs)	
Total number of Individuals	10
Sex (F/M)	9/1
Ethnicity (Caucasian/other)	10/0
Age (median/min-max)	29 (22–55)

Demographics of SLE patients	
Total number of Individuals	22
Sex (F/M)	20/2
Ethnicity (Caucasian/other)	22/0
Age (median/min–max)	42.5 (19–65)
SLEDAI-2K (median/min-max)	6.5 (2–30)
Medications used	
Cs (prednisone, cortisone)	15/22
NSAIDs (aspirin etc.)	4/22
Antimalarials (HCQ)	17/22
Immunosuppresive agents (AZA, MTX, MMF, cyclophosphamide, cyclosporine)	12/22
Biologics (Belimumab)	3/22
Clinical and laboratory characteristics	
ANA(+)	22/22
Anti-dsDNA(+)	15/22
Low complement (C3 and/or C4)	16/22
malar rash	7/22
LN	5/22

F: Female, M: Male, min-max: lowest to highest value. SLEDAI-2K: Systemic Lupus Erythematosus Disease Activity Index—2000, Cs: Corticosteroids, NSAIDs: nonsteroidal anti-inflammatory drugs, HCQ: hydroxychloroquine, AZA: azathioprine, MTX: methotrexate, MMF: mycophenolate mofetil, ANA: antinuclear antibodies, anti-dsDNA: anti-double stranded DNA antibodies, LN: lupus nephritis.

### T-bet+ B cells expand in SLE patients and correlate with disease severity

**Figure 1:**
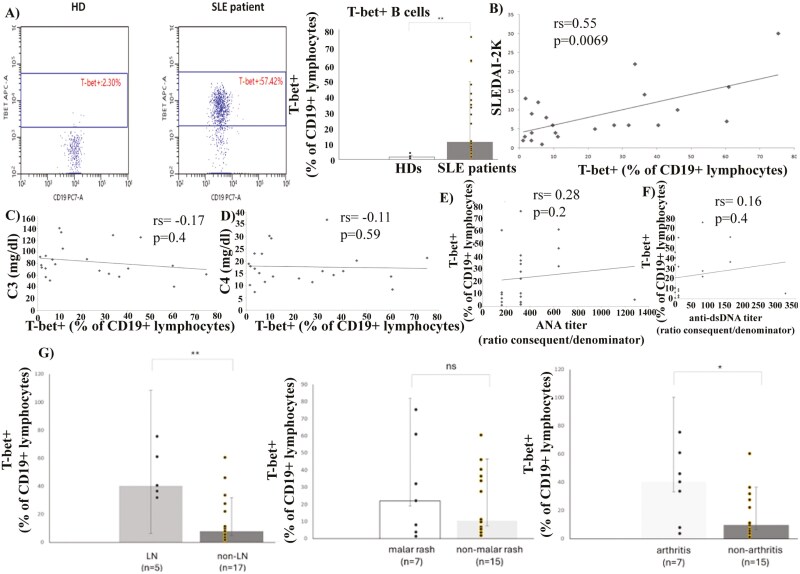
(**A**) T-bet+ B cells expand in lupus patients [1.47 (1.9–0.7) vs 10.85 (37.4–3.6)]. (**B**) T-bet B cells’ expansion correlates with SLE activity. (**C**–**F**) Weak and non-significant correlations between T-bet+ B cells and levels of C3/C4 and titers of ANA/dsDNA. (**G**) Frequencies of T-bet+ B cells correlate to LN and arthritis, in SLE patients. LN vs non-LN [40.4 (68.1–34.1) vs 7.83 (24.78–3.55)], malar rash vs non-malar rash [22(60.9–3.6) vs 10.5 (36.4–3.6)], arthritis vs non-arthritis [40.4(60.9–7.83) vs 9.7 (27.56–3.5). Data in A and G are presented as median (IQR: Q3-Q1). Correlations have been evaluated via Spearman’s coefficient. Statistical significance was evaluated via Mann–Witney’s *U* test. *<0.05 **<0.01 ns: non-significant

The flow cytometric analysis performed revealed that T-bet+ B cells displayed an expansion in SLE patients, in comparison to HDs [1.47 (1.9–0.7) vs 10.85 (37.4–3.6)] (Fig. 1A). Moreover, these cells correlated with the SLEDAI score of the patients, a factor which is indicative of disease severity (Fig. 1B) [25]. No correlations have been reported between T-bet+ B cell percentages and complement components C3 and C4 or ANA and anti-dsDNA auto-antibodies (Fig. 1C–F). We also attempted to investigate correlations between T-bet+ B cells and specific clinical manifestations, such as lupus nephritis (LN), malar rash, and/or arthritis. Interestingly, elevated frequencies of T-bet+ B cells have been observed in patients with LN and arthritis (Fig. 1G). As far as cytokines are concerned, T-bet+ B cells positively correlated with the serum levels of IFNα (no correlation was reported for TNFα) (Table 2). These circumstantial results, in total, indicate that T-bet expression in B cells may serve as a biomarker of SLE severity and may display a potential to be introduced to the clinical practice as an additional prognostic tool. Further investigations are required, involving larger cohorts of patients.

**Table 2:** correlations between T-bet+ B cell populations and the SLEDAI scores of the patients, complement components C3 and C4, ANA and anti-dsDNA autoantibodies and IFNα and TNFα cytokines

	SLEDAI	C3	C4	ANA titers	Anti- dsDNA titers	IFNα	TNFα
**T-bet+** **B cells**	rs = 0.55 *P* = 0.007	rs = -0.17 *P* = 0.4	rs = -0.11 *P* = 0.59	rs = 0.28 *P* = 0.2	rs = 0.16 *P* = 0.4	rs = 0.36 *P* = 0.05	rs = 0.05 *P* = 0.79
**ABCs**	rs = 0.3 *P* = 0.1	rs = - 0.3 *P* = 0.154	rs = -0.1 *P* = 0.6	rs = 0.02 *P* = 0.9	rs = 0.32 *P* = 0.13	rs = 0.35 *P* = 0.07	rs = 0.2 *P* = 0.33
**DN**	rs = 0.565 *P* = 0.014	rs = -0.12 *P* = 0.64	rs = -0.014 *P* = 0.95	rs = 0.22 *P* = 0.36	rs = 0.25 *P* = 0.31	rs = 0.26 *P* = 0.17	rs = 0.22 *P* = 0.28
**DN2**	rs = 0.59 *P* = 0.012	rs = -0.218 *P* = 0.4	rs = -0.044 *P* = 0.86	rs = 0.17 *P* = 0.55	rs = 0.29 *P* = 0.24	rs = 0.25 *P* = 0.19	rs = 0.18 *P* = 0.36

Statistical significant data are presented in red. rs: Spearman’s rank correlation coefficient, *P*: *P* value for statistical significance. ABCs: CD45^+^CD19^+^CD11c^+^CD21^−^T-bet^+^, DN: CD45^+^CD19^+^IgD^-^CD27^−^ T-bet^+^, DN2: CXCR5^−^ DN (extrafollicular DN).

### DN B cells (and DN2 cells) seem to be an important B cell population for SLE pathogenesis in humans

**Figure 2:**
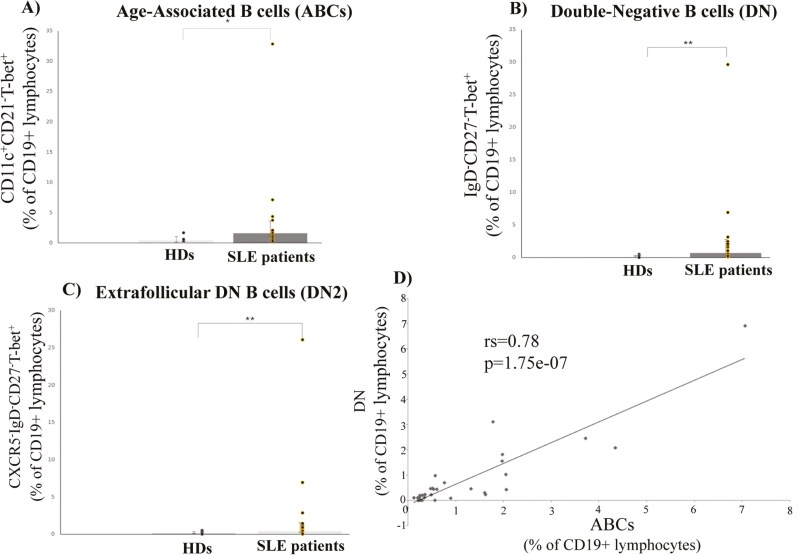
(**A**, **B**) Both ABCs and DN B cells expand in lupus patients [0.41 (0.58–0.27) vs 1.6 (2.05–0.53) and 0.1 (0.22–0) vs 0.7 (2.07–0.42), respectively (**C**) Among DN B cells, the extrafollicular subset of DN2 cells expands in lupus patients [0.1 (0.22–0) vs 0.42 (1.1–0.2)] (**D**) ABC percentages positively correlate with DN percentages. We note that a patient displaying extreme ABC-DN values, has been excluded from the correlation analysis. All data are presented as median (IQR: Q3—Q1). Correlations have been evaluated via Spearman’s coefficient. Statistical significance was evaluated via Mann–Witney’s *U* test. *<0.05 **<0.01

Among T-bet+ B cells, the sub-populations of ABCs and DN displayed a statistical significant expansion in lupus patients, when compared to the HDs [0.41 (0.58–0.27) vs 1.6 (2.05–0.53) and 0.1 (0.22–0) vs 0.7 (2.07–0.42), respectively] (Fig. 2A and B). However, only DN B cells displayed a positive correlation with the SLEDAI score of the patients (Table 2). Interestingly, among DN B cells, the extrafollicular DN2 subset also displayed an expansion in the patients [0.1 (0.22–0) vs 0.42 (1.1–0.2)] and correlated with their SLEDAI (Fig. 2C and Table 2). ABCs, on the other hand, displayed no statistically significant correlations with any of the features examined here (Table 2). Their percentages, though, positively correlated with DN percentages (Fig. 2D). It has been suggested that ABC assignment, based on the limited use of CD21, CD11c, and/or T-bet markers, integrates multiple B cell populations (including memory B cells, activated naïve B cells, and DN B cells) [26]. We do consider this scenario as the most plausible, as ABC percentages reported here were a little bit higher than DN percentages, in all the cases examined (refers to each participant).

As far as clinical manifestations are concerned, ABCs displayed no correlation to any (among LN, malar rash, and arthritis), whereas DN and DN2 subsets correlated only to LN (LN vs non-LN:2.4 (18.2–1.75) vs 0.46 (0.97–0.19), *P* = 0.004, and 1.16 (16.45–0.88) vs 0.34 (0.47–0.09), *P* = 0.01) (data not shown).

### T-bet induction and pharmacological treatments, during WBA culture experiments, had no effect on cell viability

**Figure 3:**
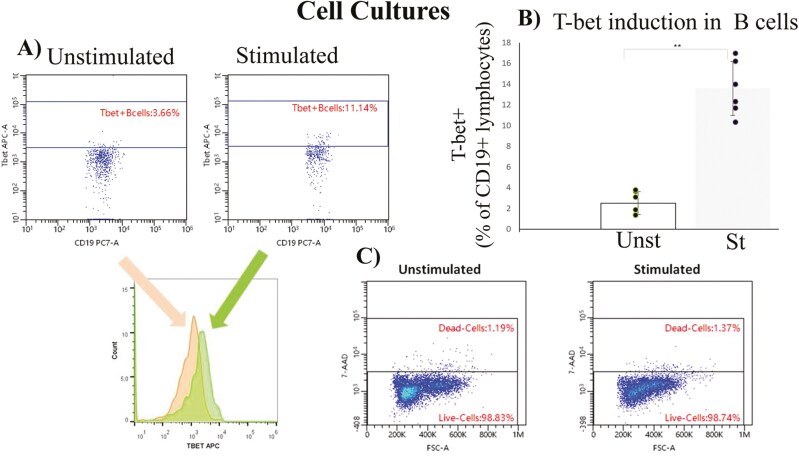
(**A)** Flow cytometry dot plots and histogram, regarding the induction of T-bet in stimulated B cells cultured. (**B**) T-bet induction in B cells cultured by synergistic triggering of TLR7, IL-21R, and IFNγR. Data have emerged from 6 independent experiments and are presented as MEAN ± SD ➔ 2.53 ± 1.1 vs 13.58 ± 2.6. Statistical significance was evaluated via Student’s *t* test. *<0.05 **<0.01 Unst: Unstimulated, St: Stimulated. (**C**) According to 7-AAD staining, there is no effect on cell viability after the stimulation of cells cultured

The stimulation of cells, in WBA cultures, has successfully induced T-bet expression in B cells (2.53 ± 1.1 vs 13.58 ± 2.6) (Fig. 3A and B). The induction of the transcription factor resulted from the stimuli used, as 7-AAD staining revealed that there was no effect on the viability of the cells cultured, following their stimulation (Fig. 3C). Moreover, the pharmacological treatments also had not affected the viability of the cells. Toxicity tests, based on the enumeration of lymphocytes, as well as of T-cell and B-cell subsets, revealed that there were no actual differences between the percentages of these populations, among all three conditions examined (unstimulated cells, stimulated only cells, and stimulated and also drug-treated cells) (Table 3).

**Table 3:** cell viability has remained unaffected, during cell culture experiments conducted.

	Lymphocytes (%)	CD3 + (%)	CD19 + (%)
UNST	41.13 ± 6	76.21 ± 2.4	13.16 ± 1.3
ST	40.8 ± 6.6	78.13 ± 1.1	12.83 ± 0.5
HCQ 1 µM	40.33 ± 8	75.66 ± 2	13.39 ± 1.2
HCQ 10 µM	41.34 ± 7.3	75.23 ± 1.7	14.04 ± 2.1
HCQ 50 µM	42.18 ± 2.95	77.53 ± 1.7	12.32 ± 1
Fas 1 µM	40.76 ± 6.4	75.58 ± 1.8	14.05 ± 2.4
Fas 10 µM	41.39 ± 3.9	77.52 ± 2.3	13 ± 1.3
Fas 50 µM	43.3 ± 3.1	77.13 ± 2.55	12.89 ± 1.4
Anif 2 µg/ml	40.26 ± 7.8	75.14 ± 2.16	14.16 ± 3.4
Anif 10 µg/ml	42.2 ± 5.7	76.2 ± 1.4	13.7 ± 1.93
Anif 30 µg/ml	41.7 ± 6.3	75.49 ± 3	15.2 ± 3.8

UNST: Unstimulated, ST: Stimulated, HCQ: Hydroxychloroquine, Fas: Fasudil, Anif: Anifrolumab. Data are presented as MEAN ± SD and have emerged from 3 independent experiments.

### T-bet+ B cells diminish, following treatment with pharmacological agents

**Figure 4:**
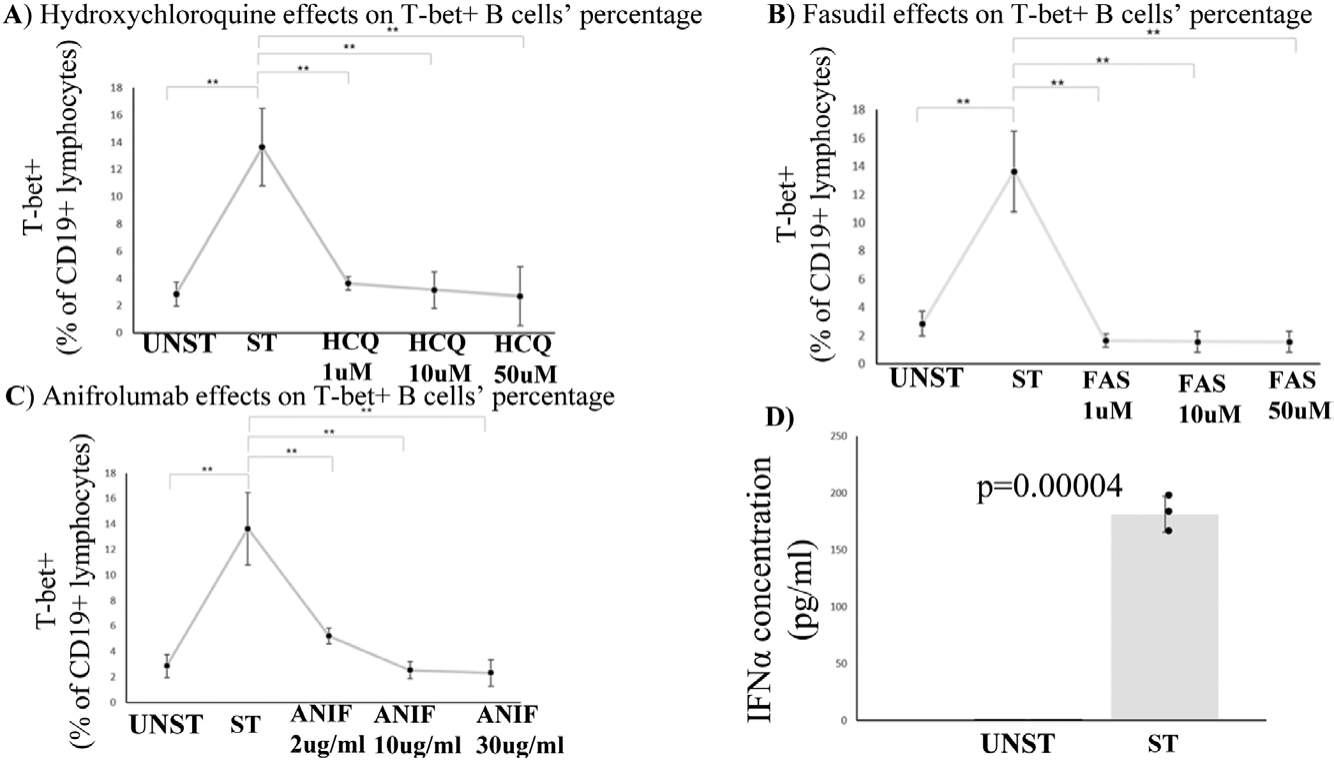
(**A-C**) T-bet + B cells diminish, following treatment with HCQ, fasudil and/or anifrolumab. Data have emerged from three independent experiments, MEAN ± SD are presented ➔ Unst: 2.85 ± 0.9 vs St: 13.64 ± 2.8 vs HCQ_1_: 3.64 ± 0.47 vs HCQ_10_: 3.15 ± 1.34 vs HCQ_50_: 2.69 ± 2.15 vs FAS_1_: 1.65 ± 0.46 vs FAS_10_: 1.57 ± 0.72 vs FAS_50_: 1.56 ± 0.74 vs ANIF_2_: 5.2 ± 0.62 vs ANIF_10_: 2.54 ± 0.68 vs ANIF_30_: 2.31 ± 1. Statistical significance was evaluated via Student’s *t* test. *<0.05 **<0.01 Unst: Unstimulated, St: Stimulated, HCQ: hydroxychloroquine, FAS: fasudil, ANIF:anifrolumab (**D**) Cell culture supernatant IFNα levels are elevated in stimulated samples, according to ELISA conducted. Data have emerged from 3 independent experiments, MEAN ± SD are presented ➔ Unst: 0.6 ± 0.01 vs St:181.3 ± 15.8. Statistical significance was evaluated via Student’s *t* test. *<0.05 **<0.01 Unst: Unstimulated, St: Stimulated

HCQ, at all concentrations tested, managed to reduce T-bet+ B cell percentages (Fig. 4A). Similarly, fasudil has led to reduced percentages of T-bet+ B cells, following its addition to the WBA cultures (Fig. 4B). Interestingly, anifrolumab has also led to diminished T-bet+ B-cell percentages in the cultures, although its effect was more dose-dependent, when compared to HCQ and/or fasudil treatments (Fig. 4C). Since anifrolumab targets the receptor of IFNα [14], and IFNα was not included as a stimulus in the cultures conducted, we hypothesized that the stimuli used in the WBA cultures, apart from inducing T-bet, also induced the cytokine. Truly, ELISA conducted for the estimation of IFNα levels in the supernatants of both unstimulated and stimulated cells cultured, revealed that the cytokine has been induced in the cultures, following the stimulation of the cells (Fig. 4D).

### According to bioinformatics analysis, two clusters of B cells highly express T-bet and display a pathogenetic role for SLE

**Figure 5:**
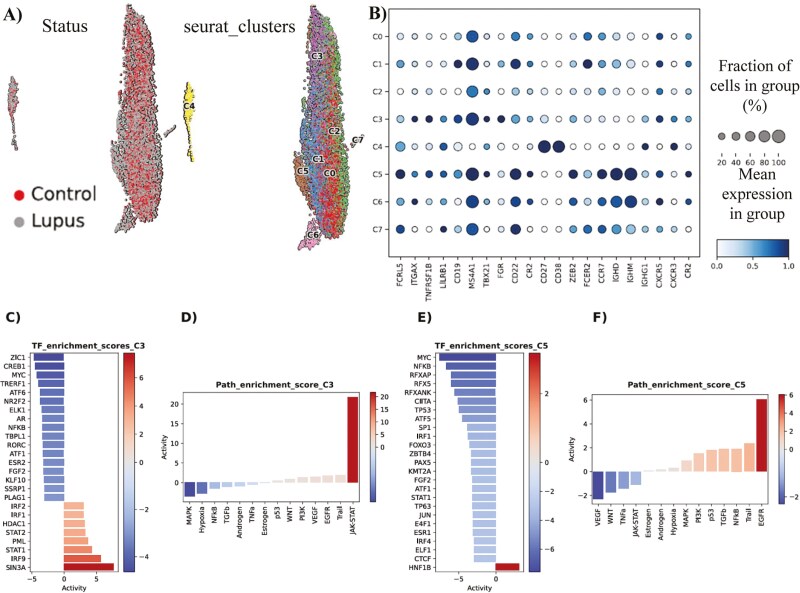
(**A)** In total, eight clusters of B cells have been identified during Seurat-based analysis. (**B**) Characterization/immunophenotyping of the eight clusters of B cells. Mean expression of key gene markers and fraction of cells in each cluster, expressing each marker, are presented. (**C**) Hyper-activated and hypo-activated transcription factors in SLE cluster 3 of B cells, in comparison to the normal counterpart cluster. (**D**) Hyper-activated and hypo-activated signaling pathways in SLE cluster 3 of B cells, in comparison to the normal counterpart cluster. (**E**) Hyper-activated and hypo-activated transcription factors in SLE cluster 5 of B cells, in comparison to the normal counterpart cluster. (**F**) Hyper-activated and hypo-activated signaling pathways in SLE cluster 5 of B cells, in comparison to the normal counterpart cluster.

Our Seurat-based analysis has identified in total eight clusters of B cells (Fig. 5A). These clusters refer to various stages of B cell differentiation, from transitional B cells to plasma cells. Interestingly, among these clusters, cluster 3 and cluster 5 have displayed elevated expression of T-bet (*TBX21*) (Fig. 5B). In more detail, cluster 3 is considered as an atypical memory B cell population, resembling ABCs due to the co-expression of T-bet and CD11c (*ITGAX*) markers. On the other hand, cluster 5 is a naïve B-cell population, highly expressing T-bet and CD11c, probably referring to activated naïve B cells (aNAV).

Following the detection of the two T-bet^hi^ B cell clusters (C3 and C5), we decided to seek underlying molecular differences in these cell populations between SLE and control samples. The differential expression analysis conducted, predicted a higher differential activity of the TFs *SIN3A*, *IRF9*, *STAT1*, *PML*, *STAT2*, *HDAC1*, *IRF1,* and *IRF2* in SLE cluster 3, compared to its control counterpart. Furthermore, an opposite trend was modeled for TFs like *ZIC1*, *CREB1*, *MYC*, *TRERF1* and *ATF6* (Fig. 5C). The ensuing Gene Regulatory Networks (GRNs), retrieved by decoupleR, revealed a tight interconnection among the various regulons of the most hyper-active TFs, with crucial target genes like *CXCL8*, *IFITM1* and *TNFSF10* to be co-regulated by more than one TF (**S6A**). Moreover, across the GRN from the regulons of the most hypo-active TFs, we detected other co-regulated gene targets (**S6B**).

In line with these findings, pathway activity analysis indicated that JAK-STAT pathway emerged as the most predominant signaling pathway in SLE cluster 3 cells, whereas the MAPK signaling pathway, along with hypoxia, exhibited notably decreased activity compared to normal cluster 3 cells (Fig. 5D). By and large, these data implicate cluster 3 cells in IFN signaling and inflammation, as well as histone modifications in SLE. Of note, T-bet seems to associate (mostly) with the hyper-activated TFs of SLE cluster 3 (**S7A-B**).

Similarly, we analyzed the cluster 5 cells and detected *HNF1B* as the only statistically significant TF with hyper-activation in the SLE cases, whereas among the various hypo-active TFs, the most prominent ones were *MYC*, *NFKB*, *RFXAP*, *RFX5*, and *TP53* (Fig. 5E). In terms of pathways, EGFR pathway was the most hyper-active signaling cascade in SLE cluster 5 cells, compared to their normal counterparts, while conversely the VEGF pathway was the most hypo-active signaling cascade (Fig. 5F). Overall, these data implicate cluster 5 cells in kidney deregulation, as *HNF1B* mutations are associated with kidney diseases and EGFR plays a major role in the development of kidney fibrosis [27, 28].

### HCQ, anifrolumab, and fasudil target hyper-active molecules in T-bet^hi^ B cells, according to *in silico* predictions

**Figure 6.**
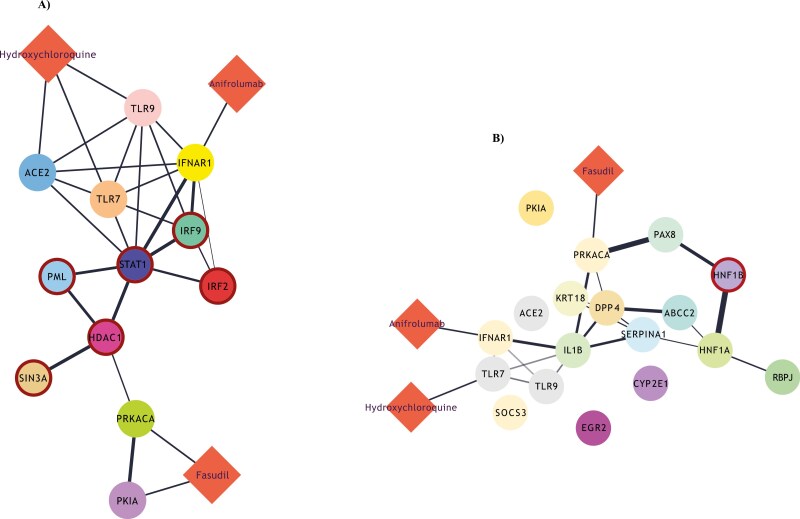
(**A)** Drug-gene bipartite network for cluster 3 of B cells, connecting the most active transcription factors of the cluster with the targets of HCQ, anifrolumab, and fasudill. (**B**) An equivalent drug-gene network for cluster 5 of B cells.

We sought to predict whether the three compounds (HCQ, anifrolumab, and fasudil), earlier tested in our *in vitro* experiments, could potentially have an impact on the most hyper-active TFs in the T-bet^hi^ B-cell clusters. To approximate this computational simulation, we constructed drug-gene bipartite networks for each cluster, which connected the most active TFs in each cluster with the targets of each drug, using STRING-db and DrugBank databases [23, 24]. Interestingly, in cluster 3 network model, we discovered that the targets of HCQ and anifrolumab (*ACE2*, *TLR9,* and *TLR7* and *IFNAR1*, respectively) were immediate neighbors of three hyper-active TFs (*STAT1*, *IRF9,* and *IRF2*), indicating a potentially important impact of these drugs to the molecular circuitries implicating these molecules. On the other hand, fasudil’s targets, namely *PKIA* and *PRKACA*, were in very close proximity with *HDAC1*, another hyper-active TF (Fig. 6A). On the contrary, in the bipartite network of cluster 5 cells, only fasudil’s target *PRKACA* was in a considerable proximity with *HNF1B*, compared to the other compounds (Fig. 6B).

## Discussion

In our study, we have put an emphasis on T-bet and attempted to investigate whether its expression in B cells can contribute to SLE prognosis and/or even therapy of the disease. A statistically non-significant difference was reported between the age of HDs and the age of SLE patients enrolled. Even so, it is important to mention that the phenomenon of age association, regarding T-bet^+^ B cells, refers mostly to splenocytes in mice [29], and is not actually present in human blood cells [3, 4, 30]. Thus, any difference between the age of HDs and the age of SLE patients should not be considered as a flaw of this study.

Firstly, in accordance with others, we detected elevated percentages of T-bet+ B cells, as well as ABCs and DN sub-populations, in SLE patients [3, 4]. Moreover, T-bet+ B cells’ percentages correlated with the SLEDAI score of the patients, a fact that indicates that these cells can probably serve as an additional prognostic marker for SLE. Similarly, the extrafollicular DN2 subset also displayed an expansion in the patients (when compared to the HDs) and correlated with disease activity. According to the literature, DN2 cells are most prominent in SLE patients of African-American ancestry [4]. However, according to our results, it seems that there is a possibility for these cells to play a role in disease pathogenesis in Caucasian patients, as well. It is important to note that ethnicity is considered as a factor that is linked to SLE manifestations and, without doubt, a better understanding of the differences among patient groups of various ancestries could probably lead to better management of the patients [31].

On the other hand, ABCs displayed no statistically significant correlations with any of the parameters examined in our study. This phenomenon coincides with the scenario of ABCs being a prominent population of B cells in mice, while their human counterparts are considered to be DN B cells [2, 32]. The truth is that consensus has not yet been reached as to whether ABCs and DN are distinct populations or just constitute the same population of B cells [26]. This is the reason why we too have treated these cells as two discrete subsets, in the first place. We attempted, though, to investigate correlations between these populations of B cells. Interestingly, ABC percentages displayed a positive correlation with DN percentages. Taking into account the fact that ABC percentages reported were a little bit higher than DN percentages, we believe that DN B cells are a subset of ABCs, with the latter cells being enriched for the former.

As far as the *in vitro* pharmacological effects tested in our study, HCQ is widely used in clinical practice for SLE cases [13], thus indubitably bringing benefits to the patients. Taking into consideration the effects of this agent on TLR signaling, regarding the inhibition of proteolytic processing of endogenous TLRs (such as TLR7) [33, 34], it is plausible to consider that HCQ administration shall have an effect on T-bet expression in B cells, as the transcription factor is strongly induced—among others—by TLR7 triggering [4, 6, 35]. Truly, our results have shown that HCQ has a direct effect on T-bet expression in B cells and successfully depletes *in vitro* this pathogenic population of lymphocytes.

Similarly, fasudil has led to reduced percentages of T-bet + B cells, following its addition to the WBA cultures. This agent refers to a ROCK kinase inhibitor that has been approved in some Asian countries for cerebral vasospam and pulmonary hypertension cases [36], and is about to be approved (probably) for other disorders, such as amyotrophic lateral sclerosis [37]. Interestingly, fasudil has attenuated lupus-like disease in lupus-prone mice models [15]. However, the main idea behind the investigation of fasudil effects on T-bet + B cells, examined in our study, stemmed from a molecular pathway, unraveled by Manni *et al*., regarding the regulation of ABCs by SWEF proteins, a two-membered family of proteins that serve as Rho-guanine enhance factors and display immunomodulatory properties [38]. Actually, in the absence of SWEF (SWAP-70 and DEF-6 proteins), transcription factor IRF5 gets deregulated and in response to IL-21 leads to the development of a lupus-like disease in mice [38]. Of note, DEF-6 is a genetic risk variant for human lupus, a fact indicating that SWEF is involved in SLE pathogenesis in humans, as well [39]. Lack of SWEF proteins, apart from leading to lupus development in mice, has also been associated with increased activity of ROCK kinase in immune cell populations [40, 41]. Taking into account all these data, we strongly believe that ROCK inhibitors may have an effect on T-bet + B cells, such as ABCs [42]. According to our results, our hypothesis turned out to be true, as all fasudil concentrations tested *in vitro* have reduced T-bet + B cell percentages.

Interestingly, anifrolumab has also led to diminished T-bet+ B cell percentages in the WBA. Of note, some studies have shown that IFNα (among others cytokines) can also induce T-bet in B cells and/or T cells [43, 44], and thus prime autoimmune diseases, such as SLE. Plasmatocytoid dendritic cells (pDCs) are considered to be the main producer of IFNα in SLE [17, 45]. These cells can be activated (along with B cells) by TLR7 agonists [46], such as the R848 agonist used in our study. ELISA conducted revealed that IFNα has been induced in the cultures, probably due to pDCs activation, thus giving an explanation to our observations regarding the effects of anifrolumab on T-bet+ B cells.

Furthermore, our scRNA-seq analysis, has detected two clusters of B cells with elevated expression of T-bet (termed as T-bet^hi^ B cells). These two clusters displayed pathogenic characteristics for SLE, such as histone modifications, hyper-activation of JAK-STAT pathway, and also kidney deregulation. The role of JAK-STAT signaling in SLE pathogenesis is well known, and the use of JAK inhibitors as potential therapeutic agents is currently under investigation [47]. It is interesting to highlight the fact that both baricitinib (a JAK1/2 inhibitor) and tofacitinib (a JAK1/3 inhibitor) treatments of mouse-isolated or human-isolated B cells have led to strong reductions of ABC/DN percentages [8], thus strengthening the scenario of JAK inhibitors being beneficial to SLE patients. In addition, EGFR pathway activation is essential for the development of kidney fibrosis, which serves as a marker of renal prognosis and also a predictor of treatment response in lupus nephritis, one of the most severe symptoms of SLE [28, 48]. As far as histone modifications are concerned, SIN3A (which serves as a subunit of histone deacetylase complex) was found to be hyper-active in T-bet^hi^ B cells. Interestingly, SIN3A is known to interact with IRF5, thus regulating the production of IFNα [49]. Of note, IRF5 is an essential transcription factor for ABC formation [38]. It is important to also mention that T-bet was found to affiliate with RORC, a transcription factor that is known to be associated with Th17 differentiation and seems to be suppressed by T-bet, in an indirect manner [50]. In accordance to the literature, RORC was found to be hypo-active in T-bet^hi^ B cells.

In addition to our *in vitro* experiments, our *in silico* analysis has suggested using HCQ, anifrolumab, and fasudil for targeting T-bet+ B cells in SLE. In more detail, HCQ seems to target TLR7 and TLR9, whose role in ABC/DN induction is of high importance [3, 4, 6, 35, 51]. ACE2, on the other hand, is a receptor associated with COVID-19 [52], a recently confronted pandemic disease that is characterized by DN expansion [53]. HCQ seems to be capable of targeting ACE2, as well. Obviously, anifrolumab targets IFNAR [14]. Interestingly, all targets of HCQ and anifrolumab were immediate neighbors of hyper-active TFs, thus indicating an impact of these drugs on the molecular circuitries implicating these aforementioned molecules. Moreover, fasudil’s targets include *PKIA* and *PRKACA*, two molecules associated with protein kinase A, whose family refers to serine-threonine protein kinases (including ROCK) [54]. Interestingly, these molecules were in close proximity with the hyper-active TFs in T-bet^hi^ B cells. These data, in total, imply that T-bet + B cells can (and probably should) be targeted for therapeutic interventions in SLE.

Lastly, our study has some limitations that are important to be mentioned. It would have been very interesting to investigate correlations between T-bet+ B-cell populations and clinical manifestations of SLE, such as lupus nephritis and/or malar rash. However, we had enrolled a limited number of patients, a fact that stemmed from the nature of medications received by the patients in our clinic (as those who were receiving cytotoxic treatment were all excluded unless 6 months had passed since the last administration of a cytotoxic agent) and also from the rarity of the disease (especially in our country, taking into account the total population) [55, 56]. Unfortunately, the small cohort of patients, in conjunction with the underrepresentation of characteristic clinical features of lupus, constitute a strong limitation to the generalizability of our results (especially given the clinical heterogeneity of the disease). Moreover, the characterization of DN2 cells in our study was based on the lack of expression of CXCR5 chemokine, as suggested by Jenks et al. who first identified this subset [4]. Truth is that we now know that the immunophenotype used here for DN2 cells (CD45^+^CD19^+^IgD^−^CD27^−^CXCR5^−^T-bet^+^) is heterogeneous, in terms of CD11c and CD19 expression statuses, as both CD11c^+^ and CD11c^-^ cells and also CD19^high^ or CD19^low^ cells are included [56–58]. The extrafollicular CD11c^-^ DN B cells have been recently identified in COVID-19, and designated as DN3 cells [57, 58], while CD19^high^ and CD19^low^ DN B cells seem to constitute two discrete populations in SLE [59]. Taking into consideration, however, the fact that DN2 cells constitute the vast majority of DN B cells in SLE [4], we believe that the extrafollicular subset examined here truly refers to DN2 cells.

The evaluation of pharmaceutical agents on T-bet+ B cells, as conducted in our study, refers to WBA cell cultures. Without a doubt, if such data had emerged from follow-up studies of SLE patients, there would have been greater importance in our results. However, patients in real life receive various medications that can strongly affect the total percentages of B cells, thus inevitably affecting the percentages of T-bet expressing B cells, as well. In addition, fasudil has not (yet) received approval for clinical use, and no clinical trials, regarding this agent in SLE treatment, are currently running [60]. To our defense, though, we note that the WBA cell culture protocol performed in our study seems to mimic well the conditions of a lupus environment, as T-bet+ B cells had been expanded and IFNα induction had been provoked, without affecting the viability of lymphocytes in the blood.

Our study focused on T-bet+ B cells, thus in the cultures conducted we aimed to induce the transcription factor, without actually inducing other ABC/DN markers, such as CD11c. The truth is that the generation and activation of these cell populations, in real conditions, derive from very complicated processes, mediated by other immune cells, such as Tfh cells [5, 6], as well as various environmental triggers, such as EBV infection [61]. Thus, to avoid any misunderstandings, we mention that we cannot guarantee that the stimuli used in our study, apart from inducing T-bet, also induced other ABC/DN-related markers.

## Final conclusions

The results of this study suggest that T-bet+ B cells, as well as DN/DN2 subset, may constitute useful prognostic tools for SLE. Furthermore, the *in vitro* and *in silico* data derived, suggest that these cells may promote lupus pathogenesis and may be targeted for therapeutic interventions. Indubitably, in order to validate all these implications, more research—involving larger cohorts of patients and additional experimental procedures—is required.

## Acknowledgements

The publication of the article in OA mode was financially supported by HEAL-Link.

## Supplementary Material

uxaf008_suppl_Supplementary_Figures_S1-S10

## Data Availability

All data are available upon reasonable request.

## Abbreviations

7-AAD: 7-amino actinomycin D
ABCs: age-associated B cells
aNAV: activated naïve B cells
ASCs: antibody-secreting cells
BCR: B-cell receptor
DN: double-negative B cells
GRNs: Gene Regulatory Networks
HCQ: hydroxychloroquine
HDs: healthy donors
LN: lupus nephritis
pDCs: plasmatocytoid dendritic cells
PBMCs: peripheral blood mononuclear cells
ROCK: Rho-associated protein kinase
scRNA-seq: single-cell RNA sequencing
SLE: systemic lupus erythematosus
TF: transcription factor
Tfh: T follicular helper cells
TLR: Toll-like receptor
WBA: whole blood assay.
