# Supplementary material for: Characterization of T-bet expressing B cells in lupus patients indicates a putative prognostic and therapeutic value of these cells for the disease
Source: Clin Exp Immunol. 2025 Feb 7;219(1):uxaf008. doi: 10.1093/cei/uxaf008 (PMC12062963; doi:10.1093/cei/uxaf008)
Supplement: uxaf008_suppl_Supplementary_Figures_S1-S10 [file uxaf008_suppl_supplementary_figures_s1-s10.docx]

| **Criteria for HDs** |
| --- |
| Subject who has not been diagnosed with an autoimmune disorder |
| Subject who has not been diagnosed with an active infection (chronic or acute) |
| Subject who does not suffer from a pathological condition, such as cancer |
| **Inclusion criteria for SLE patients** |
| Active SLE diagnosis (not necessarily a new onset) |
| Newly diagnosed SLE case |
| **Exclusion criteria for SLE patients** |
| Co-diagnosis of additional autoimmune diseases (apart from SLE) |
| Diagnosis of an active infection (such as AIDS, COVID-19 and/or hepatitis) |
| Subject suffering from cancer |
| Non-active SLE case |
| SLE cases under cytotoxic treatment (less than 6 months, since last administration) |
| Pregnant SLE patient |

**S1.** Inclusion and exclusion criteria used for the participants of the study (both healthy donors and SLE patients). All individuals were adults, older than 18 years.

**
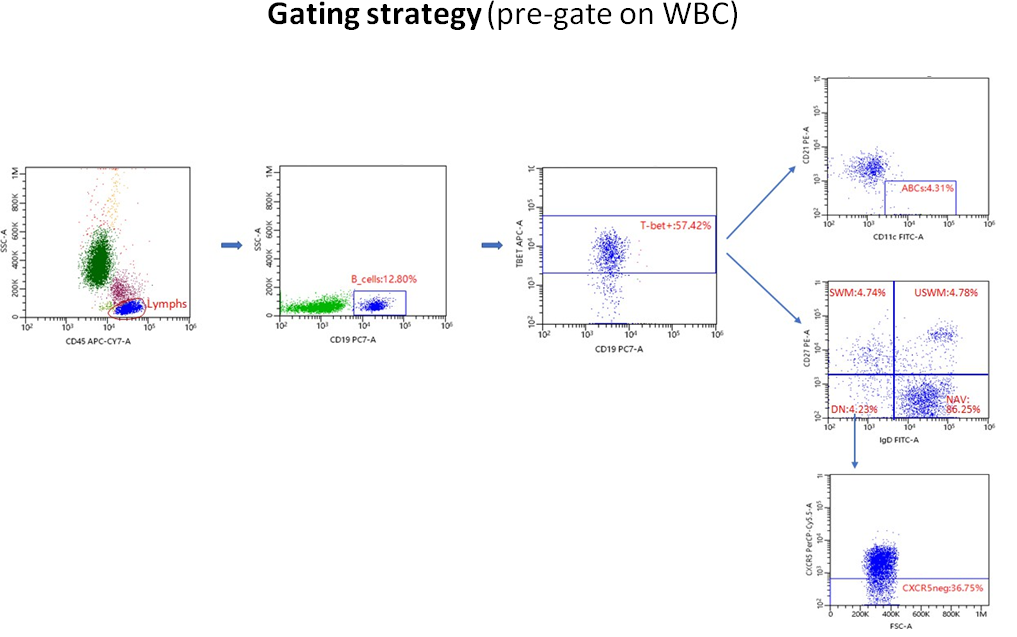
S2.** Flow cytometry gating strategy. CD45 was used for “isolating” white blood cells and gating of lymphocytes. Then, CD19 was used for the gating of B cells. We next gated T-bet+ B cells and then enumerated ABCs (as CD11c+CD21-) and DN B cells (as IgD-CD27-). In case of DN B cells, we further enumerated the extrafollicular DN B cells (termed as DN2), via CXCR5 chemokine. FSC: forward scatter, SSC: side scatter, WBC: white blood cells, NAV: naïve, USWM: un-switched memory, SWM: switched memory.

| **Basic Immunophenotype/Main Populations of Lymphocytes** | |
| --- | --- |
| Marker and Chromophore | Supplier and Clone |
| CD45 PC5 | ExBio , Clone MEM-28 |
| CD3 FITC | BioLegend, Clone OKT3 |
| CD19 PE | BioLegend, Clone SJ25C1 |
| **ABCs/DN** | |
| Marker and Chromophore | Supplier and Clone |
| CD45 APC-Cy7 | ExBio, Clone MEM-28 |
| CD19 PC7 | Beckman Coulter, Clone J4.119 |
| CD21 PE | Beckman Coulter, Clone BL13 |
| CD11c FITC | ExBio, Clone BU15 |
| IgD FITC | BioLegend, Clone IA6-2 |
| CD27 PE | ExBio, Clone LT27 |
| CXCR5 PerCP/Cy5.5 | BioLegend, Clone J252D4 |
| T-bet APC | BioLegend, Clone 4B10 |
| **Flow Cytometry Reagents** | |
| True-Nuclear^TM^ Transcription Factor Buffer Set | BioLegend (cat.# 424401) |
| Excellyse I | ExBio (cat.# ED7065) |
| Phosphate Buffered Saline/PBS 1X | Thermo Fisher Scientific (cat. #14040083) |

**S3.** Antibodies and reagents used for flow cytometry experiments.


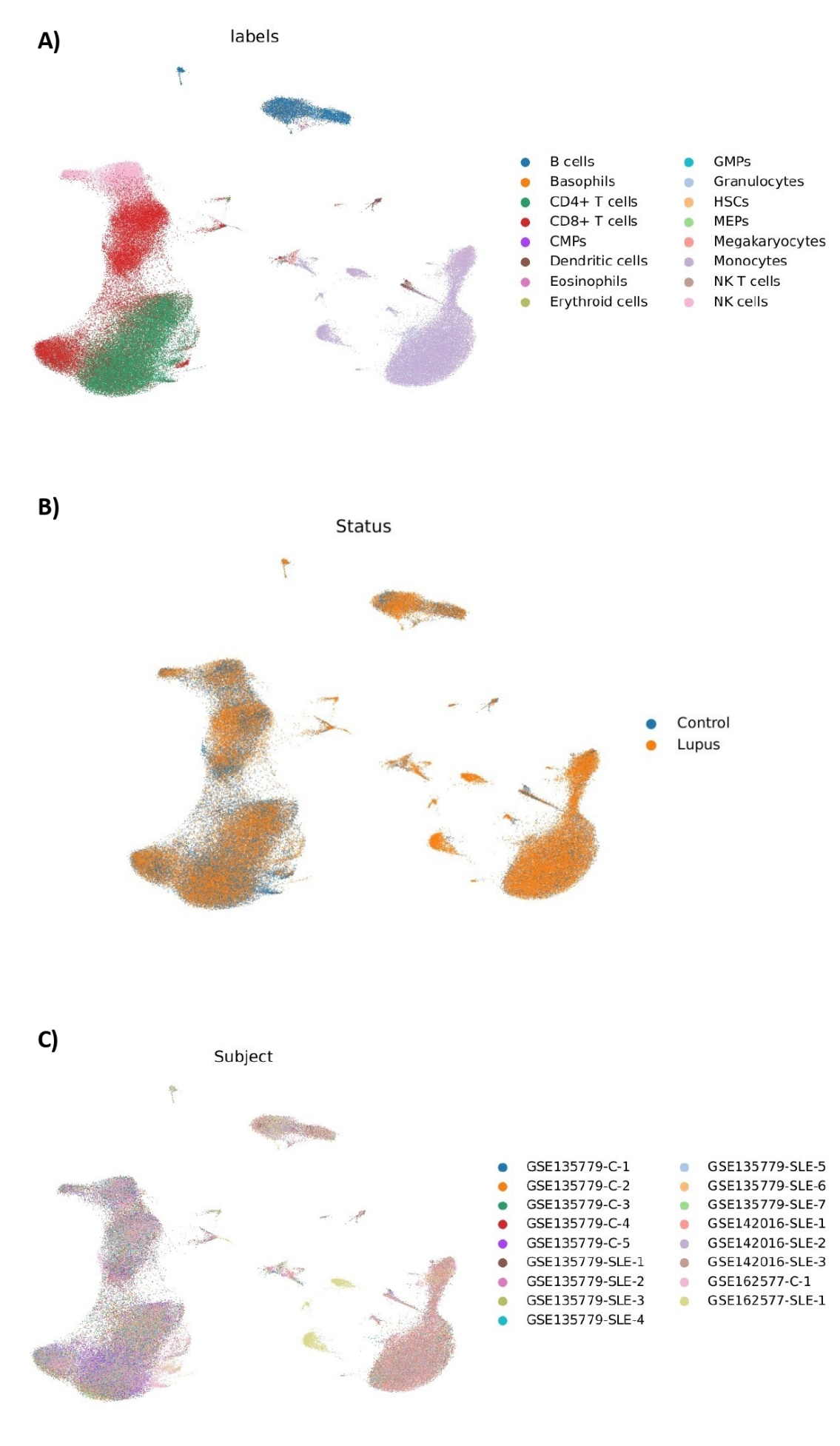


**S4.** Unified single-cell object, on the strength of (**A**) PBMC types - labels, (**B**) statuses of the individuals and (**C**) each subject.


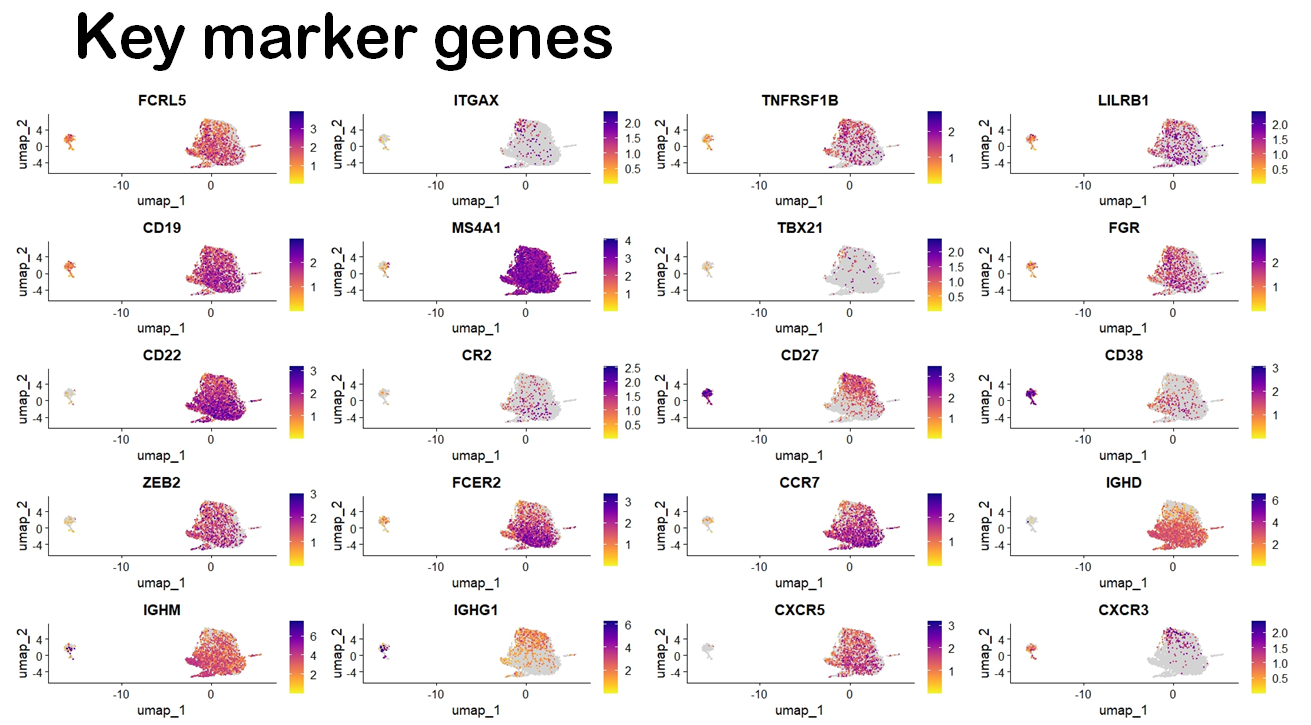


**S5.** Key marker genes used for B cell cluster identification.


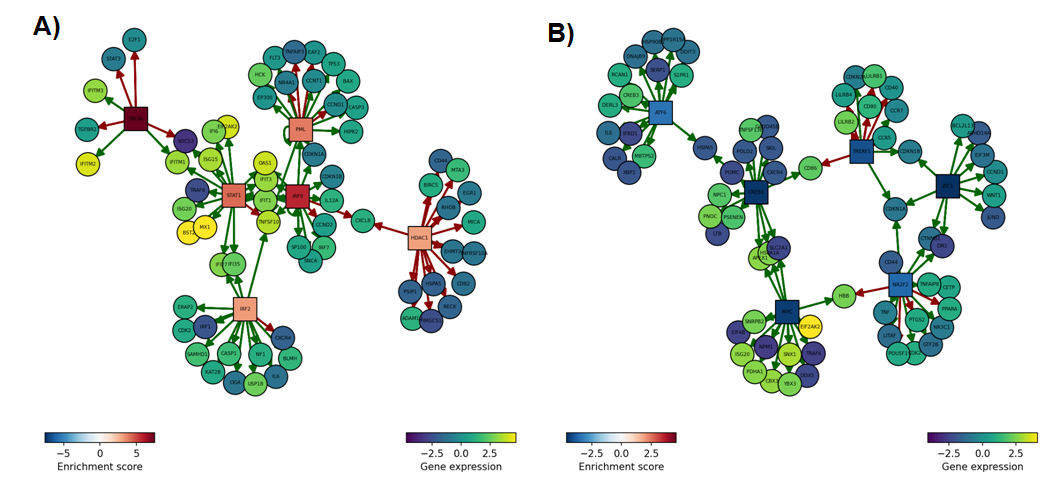


**S6.** GRNs regarding the most hyper-active (**A**) and most hypo-active (**B**) transcription factors in cluster 3 of B cells.


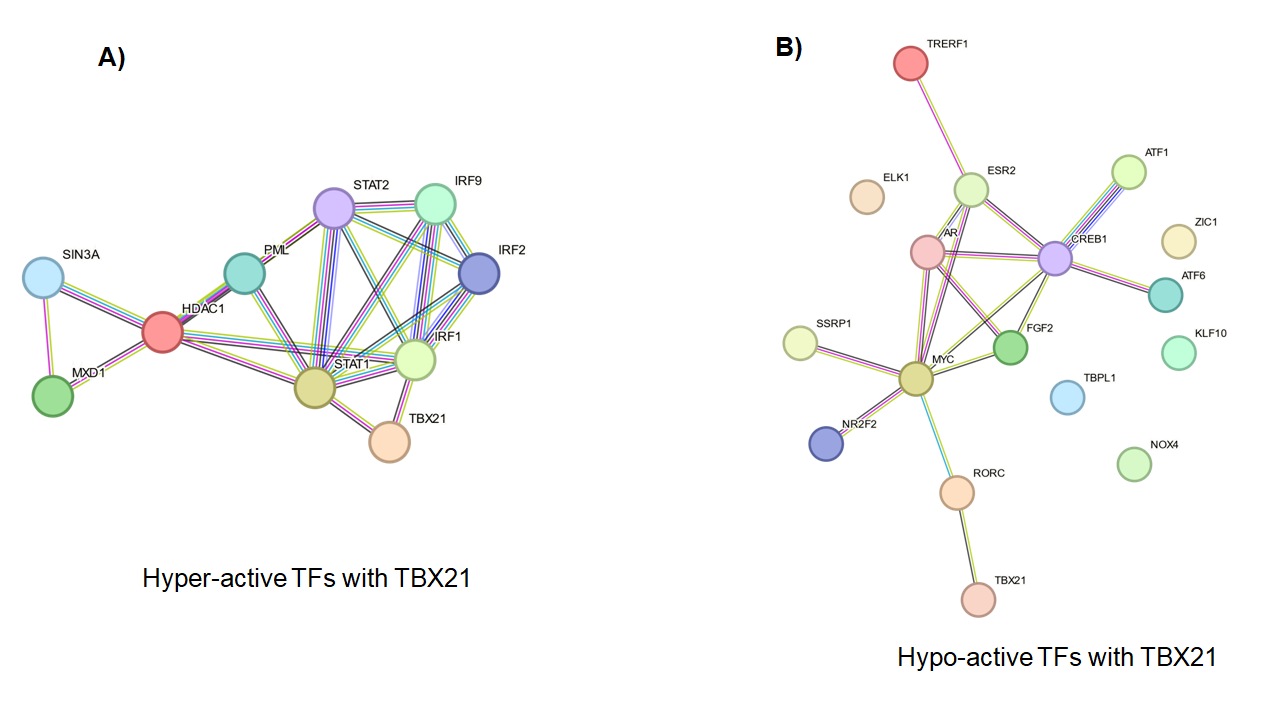


**S7. A)** T-bet (*TBX21*) affiliates with some hyper-active transcription factors, in SLE cluster 3 B cells. **B**) Among the hypo-active transcription factors, T-bet affiliates with RORC. TFs: transcription factors
